# Time Matters: One Year Later, Rethinking Anesthesia Time

**DOI:** 10.3390/jcm15145501

**Published:** 2026-07-14

**Authors:** Lucille Ridgell, José Francisco López-Gil

**Affiliations:** 1 Carver University, Orlando, FL 32804, USA; 2 School of Medicine, Universidad Espíritu Santo, Samborondón 092301, Ecuador; 3 Vicerrectoría de Investigación y Postgrado, Universidad de los Lagos, Osorno 5300000, Chile

**Keywords:** anesthesia-controlled time, healthcare policy, perioperative care, operating room efficiency, patient safety, outcome measurement

## Abstract

Anesthesia reimbursement remains anchored to time-based benchmarks, despite evidence that they poorly capture clinical complexity and variability. Recent reforms, including Utah’s 2026 ban on time limits, underscore growing policy concern. This commentary proposes replacing time-centric metrics with risk-adjusted, outcome-focused models that reflect perioperative value and patient-centered care.

## 1. Introduction

In late 2024, one of the largest health insurers in the United States proposed a policy linking anesthesia reimbursement to predefined anesthesia time benchmarks [1]. Specifically, these benchmarks were based on time estimates developed by the Centers for Medicare & Medicaid Service (CMS) rather than the actual documented duration of anesthesia care [2]. Although controversial, the proposal intended to address concerns regarding healthcare costs, payment standardization, and billing variability [3,4,5]. Historically, anesthesia reimbursement has relied on time-based metrics because anesthesia services are inherently linked to the duration of patient care, with procedure-specific time estimates incorporated into Medicare payment methodologies [2,6,7]. Within this context, proponents of benchmark-based reimbursement argued that standardized time expectations could reduce unwarranted variation, improve payment predictability, and align reimbursement more closely with typical resource utilization [3,8,9]. Similarly, studies evaluating Medicare payment systems have suggested that administrative time estimates may facilitate comparisons between observed and expected procedural durations and contribute to more consistent payment methodologies across procedures [2,10,11].

The policy, which linked reimbursement to anesthesia time benchmarks, was scheduled to take effect in 2025 and generated immediate opposition from clinicians, policymakers, and healthcare organizations, including the American Society of Anesthesiologists (ASA) [3,12]. Concerns were raised that reimbursement restrictions based on arbitrary anesthesia time limits could create financial disincentives for providers caring for higher-risk patients or performing complex procedures requiring prolonged anesthetic management [3,5]. Furthermore, opponents warned that such policies could undermine clinical decision making by prioritizing cost containment over individualized patient care and safety [3,5]. Following widespread public scrutiny and political intervention, the proposal was withdrawn [3,12,13]. One year later, the issue remains unsolved, despite ongoing policy efforts aimed at restricting the use of insurer-imposed anesthesia time caps [14,15]. This perspective piece analyzes the limitations of time-based metrics in anesthesia reimbursement and proposes a more clinically grounded approach that better reflects patient needs, procedural complexity, and the realities of perioperative care.

## 2. Key Issue in Using Anesthesia-Controlled Time

Anesthesia-controlled time (ACT) generally refers to the portions of perioperative time directly managed by the anesthesia team, including induction, emergence, and immediate operating room turnover [16,17,18]. Although ACT has long been used as an operational metric, it is shaped by numerous patient-, procedure-, and system-level factors that make it inherently variable and resistant to standardization [16,17,18]. Operating room workflow studies have shown that much of this variability is attributable to patient and procedural characteristics rather than differences in individual clinician performance [16,19]. Consequently, longer anesthesia and extubation times are frequently observed in more complex surgical procedures and among higher-risk patients [20]. While hospitals have implemented strategies such as block rooms and parallel workflows to streamline specific phases of care [21,22,23], these interventions primarily improve workflow rather than eliminate the intrinsic variability of ACT. More recently, the growing emphasis on perioperative medicine has also promoted multidisciplinary preoperative assessment, risk stratification, and patient optimization, enabling more individualized perioperative planning while improving surgical preparedness, patient safety, and coordination across the surgical pathway [24]. Importantly, these advances have demonstrated that high-quality perioperative care can enhance efficiency while maintaining a patient-centered approach rather than relying solely on reductions in ACT [25]. Nevertheless, these improvements do not always translate into meaningful gains in overall operating room efficiency, as the time saved is often insufficient to accommodate additional cases [6].

## 3. Why Current Policy Fails

The appeal of time-based reimbursement stems from the unique nature of anesthesia practice, where clinician involvement is closely tied to the duration of perioperative care [7]. Accordingly, unlike most physician services, anesthesia reimbursement has historically incorporated both procedural complexity and time units, reflecting the continuous monitoring and management required throughout a procedure [6]. From a payer’s perspective, standardized time benchmarks may improve payment predictability, reduce unwarranted reimbursement variation, and better align payments with expected resource utilization [2,3,8,10]. Such benchmarks have also been used to identify claims with anesthesia durations that substantially exceed expected procedure times. For example, a national analysis of more than six million anesthetic procedures found that practitioners with anomalous billing patterns reported anesthesia times averaging 21.5 min longer than expected [7]. However, the value of these benchmarks depends on their ability to account for clinical complexity and patient-specific factors that influence anesthetic duration; otherwise, standardized expectations may not accurately reflect appropriate perioperative care [8,11,26].

Concerns regarding the use of standardized anesthesia time thresholds have prompted increasing legislative activity at the state level since 2025 [12,15]. Following the Anthem controversy, multiple states introduced legislation to prohibit insurer-imposed anesthesia time limits and to ensure reimbursement for the full duration of medically necessary anesthesia care [27]. For example, Illinois enacted House Bill 1141 in 2025, which prohibits insurers from denying payment or reimbursement for medically necessary anesthesia services solely because the duration of care exceeds a preset time limit. The law also requires coverage for medically necessary general anesthesia regardless of its duration, with medical necessity determined by the attending anesthesiologist or licensed anesthesia provider [28]. Similar legislative efforts have been introduced across several states, including New York, Texas, Oklahoma, and Washington, reflecting a broader movement to prohibit insurer-imposed anesthesia time limits and protect reimbursement for the full duration of medically necessary anesthesia care [4]. At the national level advocacy efforts emphasized that reimbursement decisions should be based on medical necessity rather than predefined anesthesia time thresholds [4,12]. This trend continued into 2026, with Washington State reintroducing House Bill 1812, which would prohibit insurers from denying coverage or capping reimbursement based on the duration of medically necessary anesthesia services and would establish enforcement mechanisms for noncompliance [29]. More recently Utah, enacted Senate Bill 50, signed into law in March 2026, which prohibits insurers from denying payment or reimbursement for medically necessary anesthesia services based solely on preset time limitations and requires coverage for the full duration of medically necessary anesthesia care [15].

Despite these reforms, most legislative initiatives address insurer-imposed anesthesia time caps without fundamentally altering the broader reimbursement framework, in which time remains an integral component of payment [6]. Although these laws prohibit reimbursement decisions based solely on predefined anesthesia time limits, they generally preserve the use of administratively derived time benchmarks as reference points for payment and claims review [2,10,30]. Because these benchmarks are based on administrative datasets that often lack the clinical granularity needed to capture patient- and procedure-specific factors influencing anesthetic duration, they may not accurately reflect the complexity of individual cases [2,8,10,31]. Consequently, recent reforms represent a partial correction to insurer-imposed time caps by reducing the risk of payment denial or under-reimbursement while preserving a reimbursement system in which anesthesia time continues to play a central role [8].

## 4. Consequences of Duration-Based Reimbursement

The continued reliance on time-based reimbursement also raises broader concerns beyond fairness. When reimbursement systems rely heavily on duration-based metrics, they may create unintended incentives that influence clinical practice [5,6,32]. Clinicians and institutions may face financial pressures when caring for patients whose procedures require substantially longer anesthesia durations than anticipated, particularly when payment models inadequately adjust for clinical complexity [5,6,8]. Although available evidence does not suggest that anesthesia professionals routinely alter care to meet reimbursement targets, economic incentives are well established as important drivers of provider behavior across healthcare systems [32,33]. Studies evaluating physician payment models have shown that reimbursement structures can influence clinical decision-making, documentation practices, and patterns of resource utilization, underscoring the broader behavioral effects of payment design [31,32,33].

In anesthesia, such incentives may be particularly problematic because longer durations are often associated with patient and procedural complexity rather than inefficiency, underscoring the importance of adequate risk adjustment in payment models [11,26]. High-risk patients frequently require additional monitoring, careful titration of anesthetic agents, hemodynamic stabilization, and more extensive recovery assessments before transfer from the operating room [5,11,26,34]. Consequently, reimbursement frameworks that inadequately account for clinical complexity may unintentionally create incentives that disadvantage providers caring for medically complex patients or institutions serving higher-risk populations, particularly when risk adjustment is insufficient [8,31,32]. Similar concerns have been raised in other areas of healthcare, where payment systems based on simplified performance metrics may lead to unintended incentives such as risk selection, avoidance of complex patients, and prioritization of measurable targets over individualized clinical decision making [31,32,33].

These challenges are further compounded by reliance on administrative time benchmarks. Because administrative estimates cannot fully capture the patient-, procedure-, and provider-level factors influencing anesthesia duration, deviations from expected times may reflect appropriate clinical management rather than inefficiency [8,10,26]. Consequently, excessive reliance on these benchmarks may contribute to disagreements between providers and payers regarding the appropriateness of care and create reimbursement incentives that are misaligned with patient-centered, value-based care, particularly when risk adjustment is inadequate [8,31,32].

## 5. What Should Change

Reimbursement models that rely heavily on duration may fail to distinguish between inefficiency and the additional care required for high-risk or clinically complex patients [8,11,26]. An alternative approach is the use of risk-adjusted reimbursement models that account for patient, procedural, and intraoperative variability [8,26,31]. Within such frameworks, anesthesia duration is interpreted in its clinical context rather than as an isolated performance metric, while greater emphasis is placed on outcome-oriented measures such as complication rates, recovery trajectories, patient safety indicators, and adherence to evidence-based practices [8,9,11,26]. Collectively, these measures may provide a more comprehensive assessment of anesthesia care by emphasizing the quality and effectiveness of care rather than elapsed time alone, consistent with the principles of value-based healthcare [9]. However, implementing value-based reimbursement in anesthesia remains challenging because it requires robust risk adjustment, standardized outcome measures, and consistent perioperative data collection across institutions [9,35]. Until these challenges are addressed, reimbursement policies that rely predominantly on duration-based metrics may continue to generate unintended incentives that misalign payment with clinical complexity and the goals of patient-centered care [8,32].

Several anesthesia-specific alternatives to duration-based reimbursement have been proposed, including risk-adjusted payment models and approaches that incorporate clinical complexity rather than relying solely on elapsed time [8,26]. Sinclair et al. [6] developed a resource-based relative value system (RBRVS) model that preserves the time component of anesthesia services while disaggregating reimbursement into distinct phases of care, including preoperative evaluation, intraoperative management, and postoperative assessment. Unlike traditional payment structures that heavily emphasize duration, proposed frameworks recognize that anesthesia care involves a continuum of clinical decision-making and patient management extending beyond time spent in the operating room [6,8]. A review of 1195 patients across ASA physical status classifications I–V demonstrated a strong correlation between payments generated by the proposed RBRVS model and those produced under the existing ASA Relative Value Guide methodology [6]. However, reimbursement under the proposed model appeared to be more strongly influenced by clinical complexity than by procedure duration alone, with greater payment increases observed among patients requiring higher levels of anesthetic management [6]. The proposed framework generated average commercial reimbursement that was only 3.0% lower than current commercial payments, $1082 vs. $1114 per case, while Medicare reimbursement increased 2.8-fold for high complexity cases and 3.6-fold for critical complexity cases [6]. These principles are consistent with broader healthcare payment reforms that increasingly emphasize value-based, bundled, and activity-based reimbursement models designed to align payment more closely with the complexity and quality of care delivered [9,32,35].

## 6. International Approaches to Reimbursement Models

Similar principles have been adopted in healthcare systems internationally through bundled and episode-based payment models that incorporate risk adjustment for patient complexity. For example, bundled payment models in the Netherlands, Sweden, and other healthcare systems have been associated with maintained or improved quality outcomes while moderating healthcare spending growth in some settings [32,35]. In Canada, Quality-Based Procedures and bundled care initiatives incorporate case-mix adjustment to account for differences in patient acuity while reimbursing care across an episode of care, allowing flexibility in service delivery without relying on rigid limits for individual services or procedures [36]. Similarly, analyses by the Organisation for Economic Co-operation and Development (OECD) suggest that bundled and population-based payment models can account for patient complexity while promoting care coordination, efficiency, and quality of care [37,38]. Collectively, these examples demonstrate that reimbursement systems can account for clinical complexity, workload, and patient outcomes without relying primarily on rigid time thresholds [9,37]. Applied to anesthesia, such approaches would allow anesthetic duration to vary according to patient and procedural needs while shifting reimbursement toward measures that better reflect the quality, intensity, and value of perioperative care [8,26,37].

## 7. Conclusions

Overall, the evidence highlights a fundamental limitation of current approaches to anesthesia reimbursement: duration alone is an imperfect proxy for the complexity, intensity, and value of perioperative care [9,32]. Although recent policy responses, including legislative efforts to restrict insurer-imposed anesthesia time limits, reflect growing recognition of this issue, they do not address the broader structural reliance of reimbursement systems on duration-based metrics [9,32]. Consequently, administrative benchmarks continue to influence payment decisions despite substantial evidence that anesthesia duration is determined by patient characteristics, procedural complexity, and intraoperative factors that frequently fall outside the direct control of the anesthesia professional [7,26].

Beyond concerns regarding fairness, reimbursement systems that rely predominantly on duration-based metrics may create unintended incentives and fail to distinguish between inefficient care and the appropriate management of high-risk or medically complex patients [7,26]. Emerging alternatives including anesthesia-specific proposals such as the RBRVS developed by Sinclair et al. [6], demonstrate how reimbursement can capture the full scope of perioperative services while placing greater emphasis on clinical workload and management complexity. Similarly, international experiences with risk-adjusted and outcome-oriented payment models indicate that reimbursement systems can incorporate patient acuity, support quality improvement, and maintain accountability without reliance on rigid time-based thresholds [32,39].

As healthcare systems increasingly transition toward value-based care, anesthesia reimbursement models should evolve to align payment more closely with clinical complexity, quality of care, and patient outcomes rather than reliance on time-based metrics [9,32]. Future payment models should retain time as one component of service valuation while incorporating patient risk, procedural complexity, clinical decision-making, and perioperative outcomes to better reflect the full scope of anesthesia care [32,39]. Such an approach would more accurately reflect the realities of anesthesia practice, align incentives with high-quality, patient-centered care, and ensure that reimbursement better captures the true value of services provided across the perioperative continuum [9,32,39].

## Data Availability

No new data were created or analyzed in this study. Data sharing is not applicable to this article.
